# Removing a large pedunculated colonic polyp using a loop anchored with clips at the root

**DOI:** 10.1055/a-2556-6432

**Published:** 2025-03-21

**Authors:** Zhang Tao, Chao Lan, Min Huang, Tengwei Deng, Qingyu Zeng, Jie Liu, Luli Hu

**Affiliations:** 1 Department of Gastroenterology, Nanchong Central Hospital, The Second Clinical Medical College, North Sichuan Medical College, Nanchong City, China; 2 Department of Gastroenterology, Southwest Medical University, Luzhou City, China

Compared with conventional polypectomy after adrenaline injection, loop-assisted polypectomy is considered more effective and has less post-procedural bleeding in endoscopic resection of large pedunculated colonic polyps 1 . However, postpolypectomy bleeding still exists after loop-assisted polypectomy because the loop slips off the stalk or a thin stalk is transected by the loop after polypectomy 2 . We used two clips to fasten the loop at the root to avoid the stalk slipping or transecting in a large pedunculated colonic polyp. Moreover, we removed the polyp successfully without postpolypectomy bleeding, and the stalk disappeared one month later.

A 27-year-old woman presented with a large pedunculated polyp at the sigmoid colon ( Video 1 , Fig. 1 ). Two clips were used to fasten the loop ring at the root of the pedunculated polyp ( Video 1 ). We then tightened and released the loop ( Fig. 2 ). The whitened polyp was shown. A hot snare was used to entrap the root above the loop and resect the polyp intactly. The polyp measured approximately 3.5 × 3 cm ( Fig. 3 ). The white wound displayed ( Fig. 4 ). Two clips were used to close the wound. Histopathologic examination showed the large pedunculated polyp was a villous tubular adenoma without a residual tumor at the pedicle. The patient had no postpolypectomy bleeding. One-month follow-up showed the wound was clear and the pedicle had disappeared ( Fig. 5 ).

Removing a large pedunculated colonic polyp by clip-anchored loop at the root.Video 1

**Fig. 1:**
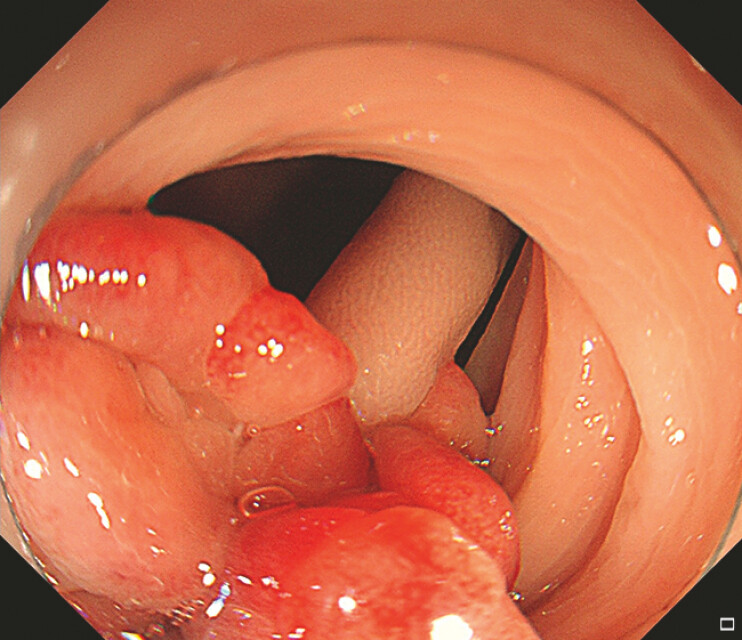
A large pedunculated polyp at the sigmoid colon.

**Fig. 2:**
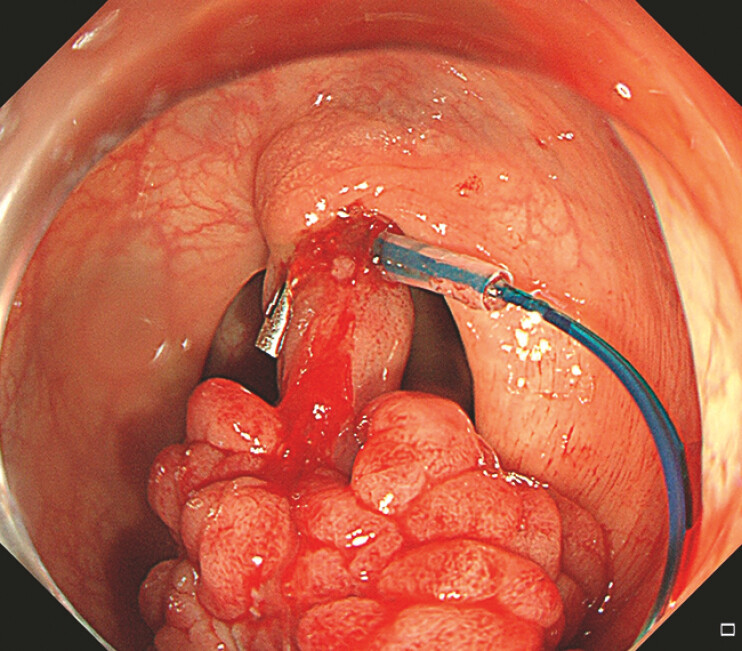
Clip-anchored loop at the root of the large pedunculated polyp.

**Fig. 3:**
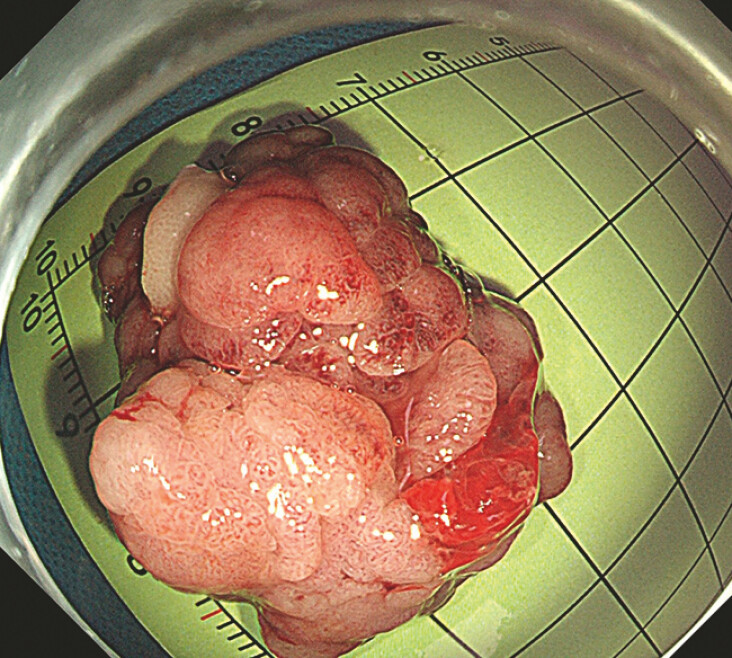
The specimen of the large pedunculated polyp.

**Fig. 4:**
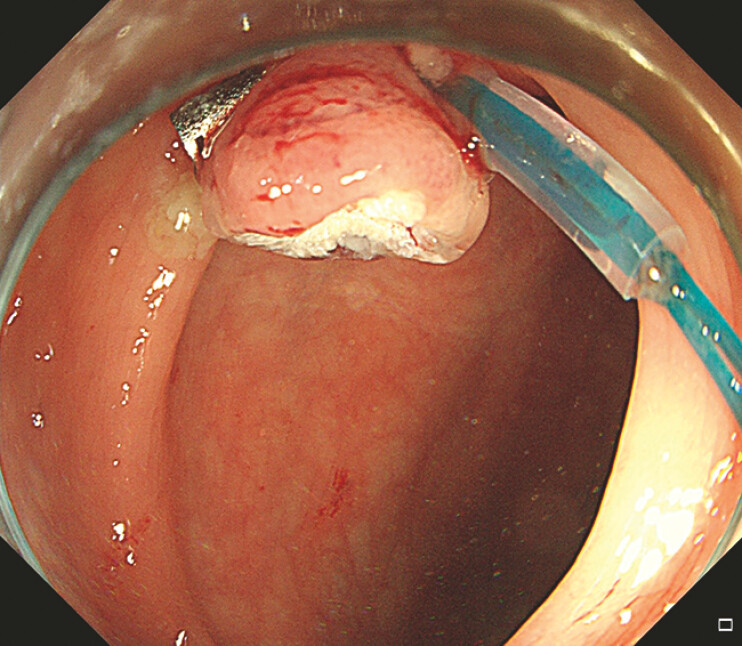
The white wound after the large polyp was removed.

**Fig. 5:**
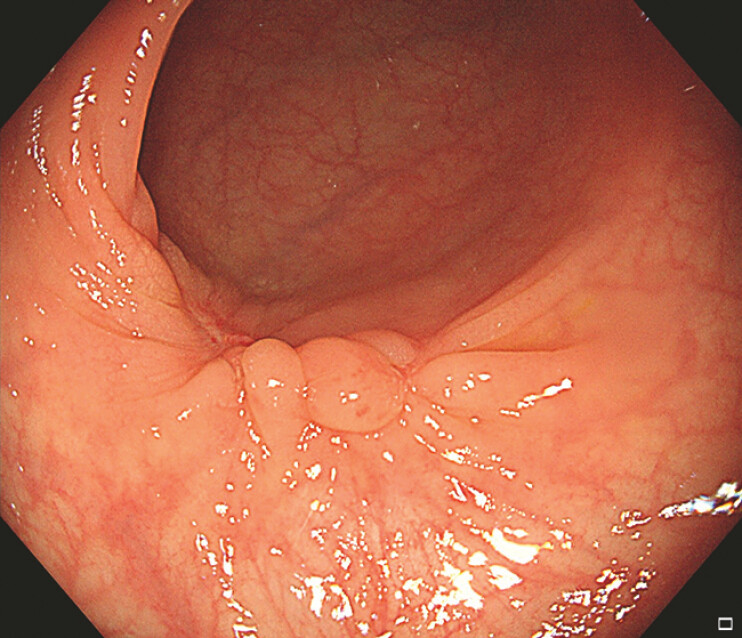
The follow-up wound with disappeared pedicle after one month.

Endoscopy_UCTN_Code_TTT_1AQ_2AD_3AB

## Notes

Endoscopy E-Videos https://eref.thieme.de/e-videos
*E-Videos* is an open access online section of the journal *Endoscopy* , reporting on interesting cases and new techniques in gastroenterological endoscopy. All papers include a high-quality video and are published with a Creative Commons CC-BY license. Endoscopy E-Videos qualify for HINARI discounts and waivers and eligibility is automatically checked during the submission process. We grant 100% waivers to articles whose corresponding authors are based in Group A countries and 50% waivers to those who are based in Group B countries as classified by Research4Life (see: https://www.research4life.org/access/eligibility/ ). This section has its own submission website at https://mc.manuscriptcentral.com/e-videos .

## References

[1] Kouklakis G Mpoumponaris A Gatopoulou A Endoscopic resection of large pedunculated colonic polyps and risk of postpolypectomy bleeding with adrenaline injection versus endoloop and hemoclip: a prospective, randomized study Surg Endosc 2009 23 2732 2737 19430833 10.1007/s00464-009-0478-3

[2] Katsinelos P Kountouras J Paroutoglou G Endoloop-assisted polypectomy for large pedunculated colorectal polyps Surg Endosc 2006 20 1257 1261 10.1007/s00464-005-0713-5 16858525

